# Cetuximab Reconstitutes Pro-Inflammatory Cytokine Secretions and Tumor-Infiltrating Capabilities of sMICA-Inhibited NK Cells in HNSCC Tumor Spheroids

**DOI:** 10.3389/fimmu.2015.00543

**Published:** 2015-11-02

**Authors:** Stephan Klöss, Nicole Chambron, Tanja Gardlowski, Sandra Weil, Joachim Koch, Ruth Esser, Elke Pogge von Strandmann, Michael A. Morgan, Lubomir Arseniev, Oliver Seitz, Ulrike Köhl

**Affiliations:** ^1^Institute of Cellular Therapeutics, Integrated Research and Treatment Center Transplantation (IFB-Tx), Hannover Medical School, Hannover, Germany; ^2^Department of Oral, Cranio-Maxillofacial and Facial Plastic Surgery, Klinikum Hanau GmbH, Hanau, Germany; ^3^Georg-Speyer-Haus Institute for Tumor Biology and Experimental Therapy, Frankfurt, Germany; ^4^Klinik I für Innere Medizin, Uniklinik Köln, Cologne, Germany; ^5^Institute of Experimental Haematology, Hannover Medical School, Hannover, Germany

**Keywords:** ADCC, cetuximab-activated NK cells, HNSCC tumor spheroids, soluble MICA, TGF-β_1_

## Abstract

Immunosuppressive factors, such as soluble major histocompatibility complex class I chain-related peptide A (sMICA) and transforming growth factor beta 1 (TGF-β_1_), are involved in tumor immune escape mechanisms (TIEMs) exhibited by head and neck squamous cell carcinomas (HNSCCs) and may represent opportunities for therapeutic intervention. In order to overcome TIEMs, we investigated the antibody-dependent cellular cytotoxicity (ADCC), cytokine release and retargeted tumor infiltration of sMICA-inhibited patient NK cells expressing Fcγ receptor IIIa (FcγRIIIa, CD16a) in the presence of cetuximab, an anti-epidermal growth factor receptor (HER1) monoclonal antibody (mAb). Compared to healthy controls, relapsed HNSCC patients (*n* = 5), not currently in treatment revealed decreased levels of circulating regulatory NK cell subsets in relation to increased cytotoxic NK cell subpopulations. Elevated sMICA and TGF-β_1_ plasma levels correlated with diminished TNFα and IFN-γ release and decreased NKG2D (natural killer group 2 member D)-dependent killing of HNSCC cells by NK cells. Incubation of IL-2-activated patient NK cells with patient plasma containing elevated sMICA or sMICA analogs (shed MICA and recombinant MICA) significantly impaired NKG2D-mediated killing by down-regulation of NKG2D surface expression. Of note, CD16 surface expression levels, pro-apoptotic and activation markers, and viability of patient and healthy donor NK cell subpopulations were not affected by this treatment. Accordingly, cetuximab restored killing activity of sMICA-inhibited patient NK cells against cetuximab-coated primary HNSCC cells via ADCC in a dose-dependent manner. Rapid reconstitution of anti-tumor recognition and enhanced tumor infiltration of treated NK cells was monitored by 24 h co-incubation of HNSCC tumor spheroids with cetuximab (1 μg/ml) and was characterized by increased IFN-γ and TNFα secretion. This data show that the impaired NK cell-dependent tumor surveillance in relapsed HNSCC patients could be reversed by the re-establishment of ADCC-mediated effector cell activity, thus supporting NK cell-based immunotherapy in combination with antineoplastic monoclonal mAbs.

## Introduction

Natural killer cells are lymphoid effector cells important for the innate immune response against virally infected and malignant cells (1, 2). NK cells eliminate transformed target cells during cytotoxic interactions by releasing pro-inflammatory cytokines, especially IFN-γ and TNF-α (3). Similarly, tumor-infiltrating NK cells can trigger stimulating interactions via “cell cross-talk” with dendritic cells (DCs), possibly facilitating tumor antigen presentation and induction of tumor antigen-directed T-cell responses. This demonstrates the constitutive role of NK cells as mediators between the innate and acquired immune systems (4–6). In this respect, NK cell killing activity is regulated by both stimulatory and inhibitory receptors. Interaction between NK cell inhibitory receptors in the presence or absence of MHC class I molecules on normal and possible target cells was described as the “missing self” hypothesis (7, 8). Activating receptors include the natural cytotoxicity receptors (NCRs) NKp30, NKp44, and NKp46 with poorly characterized ligands, and the NKG2D receptor, which recognizes a variety of well-defined ligands expressed by transformed cells (9–12). Several studies confirmed the predominant relevance of NKG2D in efficient recognition and elimination of tumor- and “stressed” cells by targeted binding of MICA and MICB (9, 10, 12). Interestingly, elevated levels of soluble forms of these NKG2D ligands (sMICA and sMICB), generated by matrix metalloproteinase (MMP)-dependent proteolytic cleavage (“shedding”) were detected in plasma/serum of cancer patients (13). These soluble NKG2D ligands are responsible for systemic reduction of NKG2D expression on the surface of various circulating blood lymphocytes, especially cytotoxic NK cells, NK-like T (NKT) cells, and CD8^+^ αβ^+^- and γδ^+^-T cells. Thus, these immune modulating effects resulted in decreased tumor surveillance by attenuated recognition and elimination of malignant cancer cells (14–16).

Some reports described NK cell dysfunction in patients with head and neck squamous cell carcinoma (HNSCC). These highly aggressive solid tumors originate from the epithelial lining of the upper aero-digestive tract and are able to escape NK cell-mediated immunosurveillance. Tumor progression is accomplished by significantly reduced expression levels of NKG2D on effector cells (17–20). Detection of increased sMICA plasma levels monitored in HNSCC patients at advanced disease stages (stage IV) and poor clinical prognosis further supports the importance of diminished tumor surveillance in HNSCC progression (21, 22). Indeed, high sMICA levels coincide with increased frequencies of lymph node (LN) metastasis. Additionally, decreased survival rates in high-risk cancer patients are potentiated by high sMICB levels (19, 20, 23). Interestingly, the multiple dysfunctions of NK cells can be largely reversed by cancer antigen-targeted antibodies, which stimulate the antibody-dependent cellular cytotoxicity (ADCC)-mediated cytotoxicity of activated NK cells to selectively eliminate malignant cells. Therefore, multiple monoclonal antibodies (mAbs) have been designed to target diverse tumor surface molecules. Based on the assumption that mAbs interact specifically with tumor target molecules, mAbs could affect tumor cells by directly inhibiting essential signaling pathways initiated by target molecules and/or by stimulating effector cell cytotoxicity, resulting in tumor elimination. One highly investigated cancer antigen for treatment of solid tumors is the epidermal growth factor receptor (EGFR). EGFR is a member of the ErbB protein family, which consists of four transmembrane receptor proteins, including HER1 [EGFR, ErbB1: avian erythroblastic leukemia viral (v-erb-b) oncogene homolog, receptor for EGF], HER2 (ErbB2), HER3 (ErbB3), and HER4 (ErbB4) (24–26). EGFR overexpression is associated with poor clinical prognosis in multiple solid tumors and EGFR signaling plays an important role in malignant cell migration, evasion, and proliferation (27). Therefore, mAbs designed to target EGFR were developed. In the last decade, cetuximab, an anti-EGFR human-mouse chimeric IgG_1_ monoclonal antibody (mAb) was approved by the Food and Drug Administration (FDA) for treatment of metastatic colorectal cancer, metastatic non-small cell lung cancer, and HNSCC patients (28, 29). However, only a weak to moderate (10–20%) benefit was observed in clinical trials with high-risk cancer patients (30–35). In this context, HNSCC cells may have evaded NK cell immunosurveillance due to polymorphisms in the FcγRIIIa (CD16a) on effector cells that impact interaction with the IgG_1_ Fc, heavy-chain, portion of cetuximab. This partially elucidates patient-specific responses to cetuximab and underscores the essential importance of modulating immunological synapses (36–38). In addition, the tumor microenvironment can impact lymphocyte-dependent immunosurveillance, which correlated strongly with tumor infiltration as well as NK cell-mediated killing activity. The ability to control these factors could contribute to improved prognosis in some malignant diseases (39–42). Thus, NK cells as key players in ADCC-related cetuximab activity were able to infiltrate primary colorectal adenocarcinomas and NK cell infiltration was an independent predictor for response and progression-free survival in patients receiving cetuximab treatment (43).

Recently, we described a decreased anti-tumor recognition, cytokine release and a reduced NKG2D expression on NK cells from untreated HNSCC patients. *In vitro* blocking experiments revealed a synergistic negative effect of sMICA potentiated by TGF-β_1_ on the killing activity of patient NK cells (22). In the current study, cetuximab treatment reconstituted the tumor surveillance capacity of sMICA-inhibited NK cells from HNSCC patients (*n* = 5), thus demonstrating the potential usefulness of cetuximab in retargeted ADCC. In order to investigate specific NK cell-dependent tumor infiltrations and ADCC-related cetuximab response, we developed HNSCC tumor-like cell clusters and a tumor spheroid model derived from primary, singularized tumor cells from these HNSCC patients. Our results indicate a crucial relevance of enhanced sMICA levels in tumor surveillance and infiltrations of inhibited patient NK cells. Finally, we demonstrate that these immunosuppressive effects on NK cell-mediated killing activity could be bypassed using cetuximab-coated HNSCC cells.

## Patients and Methods

### HNSCC Patients

**Figure 1:**
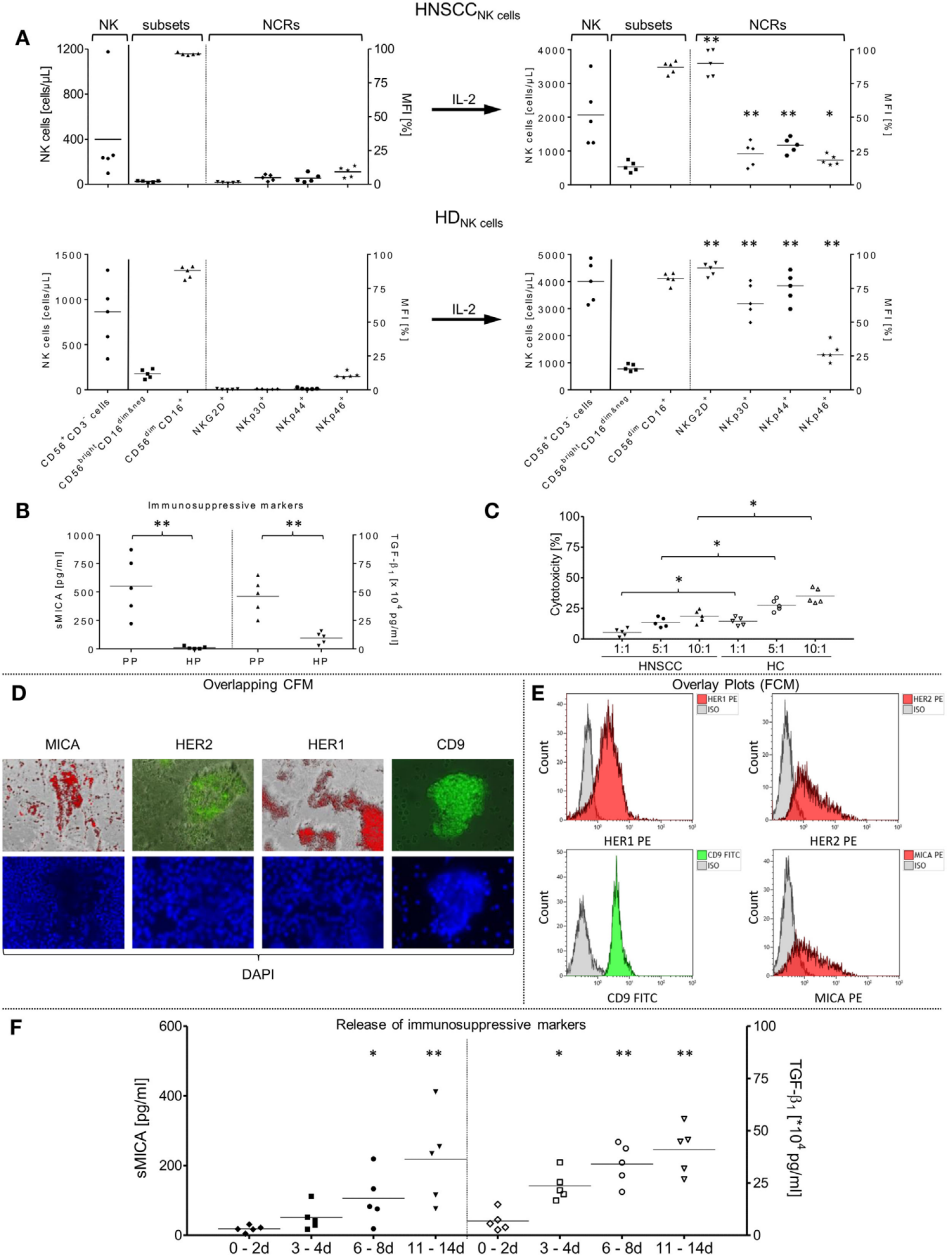
**Phenotypical and functional characterizations of patient NK cell subsets and primary tumor cells from non-treated HNSCC patients (*n* = 5) after tumor relapse**. **(A)***In vitro* rearrangement of the NK cell phenotype was quantified in the PB before separation of NK cells and after IL-2 expansion (1000 IU/ml IL-2; 9–12 days). Shown are the absolute numbers of patient (HNSCC_NK cells_) and healthy donor (HD_NK cells_) CD56^+^/CD3^−^ NK cells [cells/μl] [left graph area (NK)], the mean fluorescence intensity [MFI (%)] of distribution of resultant CD56^bright^/CD16^dim&neg^ and CD56^dim^/CD16^+^ NK subpopulations [middle graph area (subsets)] and co-expressed NCRs [MFI (%) right graph area (NCRs)] among total NK cells. **(B)** SMICA and TGF-β_1_ levels were analyzed in blood plasma from corresponding HNSCC patients (PP) and compared to age-matched healthy donor plasma controls (HP). **(C)** Assessment of the basic killing activities between effector cells isolated from patient and healthy donor NK cells against SCC-4 target cells. Freshly isolated, non-stimulated NK cells from patients (HNSCC), and healthy controls (HC) were treated with corresponding HNSCC patient plasma (high sMICA/TGF-β_1_) or associated healthy control plasma (low sMICA/TGF-β_1_) and co-incubated for 4 h (37°C, 5% CO_2_, 250 rpm) with SCC-4 cells at the indicated E:T ratios and cytotoxicity (%) was measured by FCM. **(D,E)** Immunofluorescence staining and FCM-based characterization of relevant tumor antigen expression from primary tumor samples derived from corresponding HNSCC patients (*n* = 5). After preparation of single cell suspensions from primary tumor samples, tumor cells were cultured (1–2 days, 37°C, 5% CO_2_) on chamber slides and characterized phenotypically for CD9, MICA, HER1, and HER2 expression profiles by immunofluorescence microscopy. Depicted is the staining for one representative HNSCC sample. **(D)** The HNSCC cells were also analyzed for CD9 (FITC), MICA (PE), HER1 (PE), and HER2 (PE) surface expression levels by FCM **(E)**. Nuclei were stained with DAPI (4′,6-diamidino-2-phenylindole, blue fluorescence). **(F)** Secretion of soluble immunosuppressive factors derived from five tumor samples. SMICA and TGF-β_1_ levels in expanded primary HNSCC cell cultures were analyzed by ELISAs in the collected cell medium supernatants at the indicated time frames. **(A–F)** Data are shown as mean ± SD from two to four experiments for the five patients. Range of statistically significant differences: from *p ≤ 0.01 to **p ≤ 0.001.

We analyzed five HNSCC patients (three male and two female, age range: 24–76 years) and five age-matched healthy individuals (three male and two female, age range: 26–58 years) served as controls. Histopathology confirmed that patients had stage II–IV HNSCC. Patients were included in this study after tumor recidivism but before initiation of any clinical treatment (Table 1). Corresponding patient blood samples (80–100 ml) were received shortly before the tumor surgery and associated tumor fragments were collected during tumor extractions from all patients. Informed consent was obtained from patients, caretakers, and healthy controls (HCs). Patient characteristics are summarized in Table 1. Blood samples were collected from HNSCC patients and healthy individuals in Heparin- and EDTA-coated tubes. Total leukocytes and the resultant subpopulations were counted by five-color flow cytometry (FCM) analysis as described previously (44). Immunocompetent cell subpopulation distributions were compared among patients and HCs (Figure 1).

**Table 1:** **The clinical parameters and immune status of HNSCC (*n* = 5) patients summarized after tumor (TU) and lymph node (LN) surgery but before any acute clinical therapeutic regimens**.

Patient characteristics		1	2	3	4	5
Age (years)		24	69	66	76	29
TNM classification		T2N2M1	T3N2M1	T3N2M1	T1N1M0	T2N2M0
Grading		2	2	2	1	2
Treatment/chemotherapy		Surgery/−	Surgery/−	Surgery/−	Surgery/−	Surgery/−
TU/LN locations		Neck/ear	Upper gingiva/lower jaw	Neck/ear	Jaw angle	Tongue
TU/LN material for research		+/−	+/+	+/+	+/+	+/−
**Immune status**	Leukocytes (cells/μl)	11,000	6250	8000	4900	8200
	Lymphocytes (% of leukocytes)	13.6	19.4	14.6	11.5	43.2
	Monocytes (% of leukocytes)	10.5	4.5	8.9	6.6	6.2
	T cells (% of lymphocytes)	74.7	70.1	82.7	82.2	54.9
	B cells (% of lymphocytes)	11.2	1.9	12.1	6.2	5.0
	NKT cells (% of lymphocytes)	1.1	7.5	1.7	1.3	3.4
	NK cells (% of lymphocytes)	12.8	19.5	2.7	9.3	33.2

### Target Cell Line

The human HNSCC cell line SCC-4 (ATCC: CRL-1624) (45, 46) was used to compare the cytotoxic activities of freshly purified patient and healthy NK cells and served as an internal control for scored intensities of comparable fluorescence staining’s from different patient-derived primary HNSCC cells. Therefore, the SCC-4 was cultured in DMEM and GlutaMAX™ medium (GIBCO, Invitrogen, Germany) supplemented with 10% (v/v) heat-inactivated fetal bovine serum (FBS) and 2 mM l-glutamine (PAA Laboratories GmbH, Austria).

### Preparation of Single Cell Suspension from Primary Tumor Samples

Tumor samples from untreated HNSCC patients (*n* = 5) were collected post-surgery and washed twice in serum-free DMEM/F12HAM/Glutamax supplemented with 100 U/ml penicillin, 100 μg/ml streptomycin, 0.25 μg/ml amphotericin B (antibiotic–antimycotic 100×, all purchased from Life Technologies, Gibco^®^, Darmstadt, Germany). After dissociation with 0.05% trypsin/EDTA (Life Technologies, Gibco^®^, Darmstadt, Germany), tumor pieces were minced with scissors and scalpels in a sterile dish. The digestion was stopped with DMEM/F12HAM/Glutamax containing 10% AB-Serum (former: PAA, Linz, Austria), antibiotic–antimycotic and the sample was passed through a 70 or 100-μm nylon mesh cell strainer (BD Biosciences, Heidelberg, Germany) to achieve single cell suspensions. Cells were collected in a 50-ml conical tube and subsequently centrifuged. Suspended cells were counted with trypan blue, characterized with FCM and cultivated in DMEM/F12HAM/Glutamax/10% AB serum/antibiotic–antimycotic in an incubator (37°C, 5% CO_2_, 90% humidity). Cultured tumor cells formed small tumor cell clusters after a few days, which resulted in tightly arranged HNSCC tumor spheroids (diameter: 1–3 mm) after cell cultivation of 1–2 weeks. Tumor cluster and spheroids derived from our HNSCC patients were used for NK cell-based cytotoxicity and tumor-infiltration assays monitored by fluorescence microscopy and time-lapsed transmitted imaging.

### Cytokine Analysis

The BD CBA Kit was utilized for scavenging soluble cytokines, especially IFN-γ and TNFα with beads of known size and fluorescence, allowing identification of soluble molecules in blood or supernatants from cell culture medium by FCM as described previously (44).

### Quantification of sMICA and TGF-β_1_ in HNSCC Patients

The *BAMOMAB* MICA-Sandwich ELISA kit for sMICA (AXXORA GmbH, Germany) was designed for quantification of soluble MICA (sMICA). The kit was utilized for detection and monitoring of immunosuppressive molecules in HNSCC patient blood plasma (*n* = 5), HCs (*n* = 5), and supernatants of cell culture medium during adherent growth phase of tumor cell clusters and generation of tumor spheroids as described previously (47). TGF-β_1_ levels in human blood plasma samples and cell culture supernatants were quantified by MTPL ELISA (Milenia Biotec, Version 3.0, Germany).

### Immunomagnetic Separation of CD56^+^CD3^−^ NK Cells

Up to 100 ml heparinized blood from HNSCC patients (*n* = 5) was used to isolate viable mononuclear cells (MNC) in high yield and purity by Ficoll-Paque density gradient. Primary NK cells were separated from purified MNCs via “non-touched” depletion using the EasySep^®^ Human NK Cell Enrichment Kit (STEMCELL Technologies SARL, Germany). Other leukocyte subsets were labeled with tetrameric antibody complexes against CD3, CD4, CD14, CD19, CD20, CD36, CD66b, CD123, HLA-DR, glycophorin A, and dextran-coated magnetic particles. The non-labeled cells were scavenged by an EasySep^®^ hand magnet according to the manufacturer’s recommendations. Freshly purified NK cells (purity: 95.1 ± 2.8%) were expanded and activated with 1000 IU/ml IL-2 for 9–12 days as described previously (47).

### Production of Shed MICA

To generate shMICA, the DNA sequence encoding for full length MICA engineered with an N-terminal histidine-tag was cloned into a tet-on vector system and transfected into the UKF-NB3 tumor cell line using the Neon transfection system (Life Technologies, USA). Cells were selected with 0.25 mg/ml G418 and 1 μg/ml puromycin (Life Technologies, USA). MICA expression was induced by addition of 2 μg/ml doxycycline (Sigma, Germany). Further on cells were stressed by serum starvation for 72 h to induce MICA shedding. Finally shMICA was purified with Protino Ni-NTA agarose (Macherey-Nagel, Germany) from cell culture supernatants and concentrated using Amicon centrifugal filter units (Merckmillipore, Germany).

### Cytotoxicity Assays

**Figure 2:**
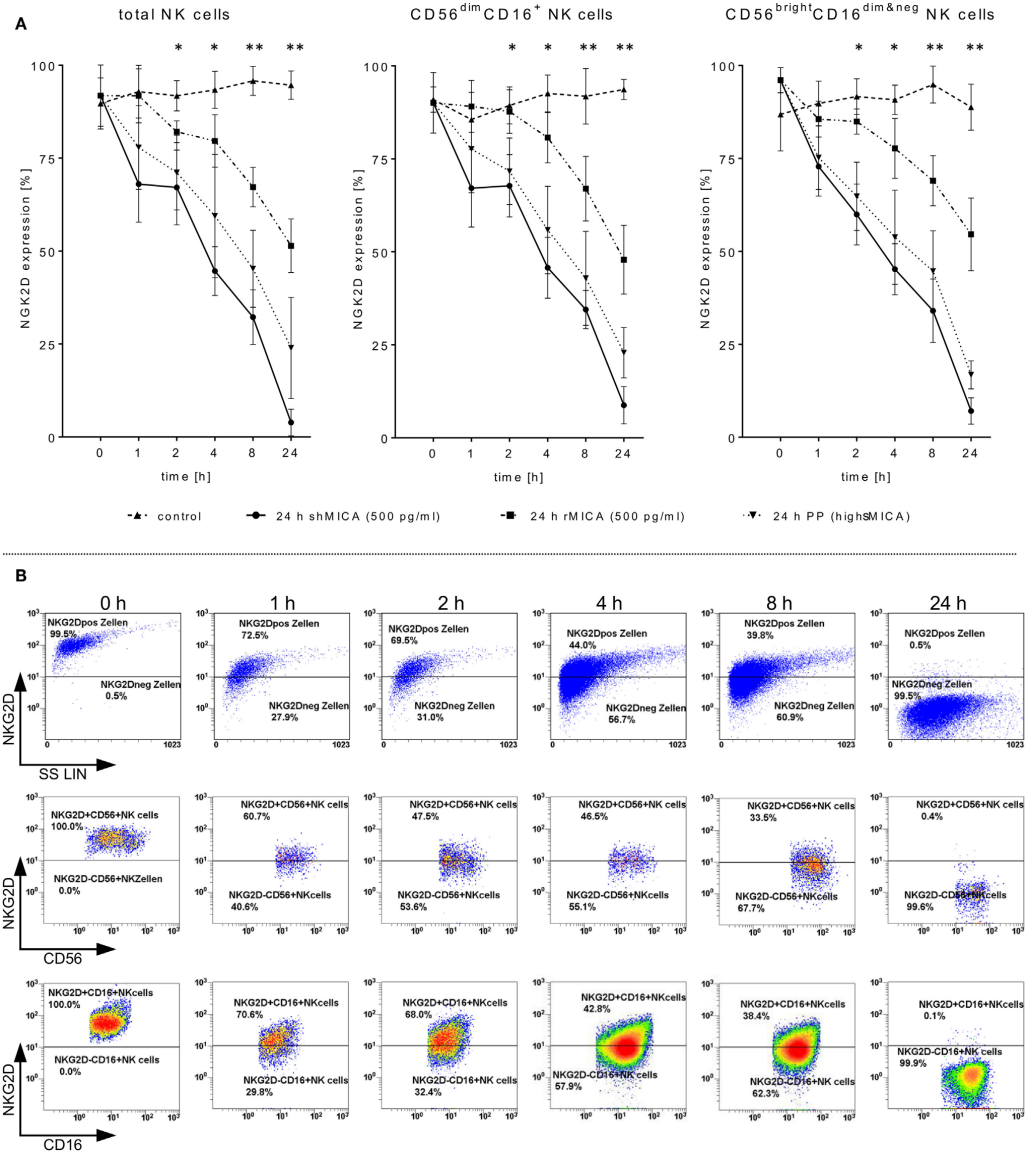
**Impact of sMICA on NKG2D surface expression and NKG2D-mediated NK cell cytotoxicity against HNSCC cells**. Isolated CD56^+^/CD3^−^ NK cells from untreated HNSCC patients (*n* = 5) were stimulated for 9–12 days with 1000 IU/ml IL-2. **(A)** Effect of high sMICA levels on NKG2D surface expression. IL-2 activated patient NK cells were incubated (37°C, 5% CO_2_) at the indicated points of time with shMICA (500 pg/ml), rMICA (500 pg/ml) and patient plasma containing high MICA levels (PP, range: 220.9–870.7 pg/ml). The NKG2D expression levels on total NK cells and both CD56^dim^/CD16^+^ and CD56^bright^/CD16^dim&neg^ NK subsets were compared to non-treated control NK cells. **(B)** Phenotypical analyses on time-dependent impact of shMICA on NKG2D surface expression were determined by FCM. Exemplarily depicted here are representative dot plots for one time-dependent experiment.

**Figure 3:**
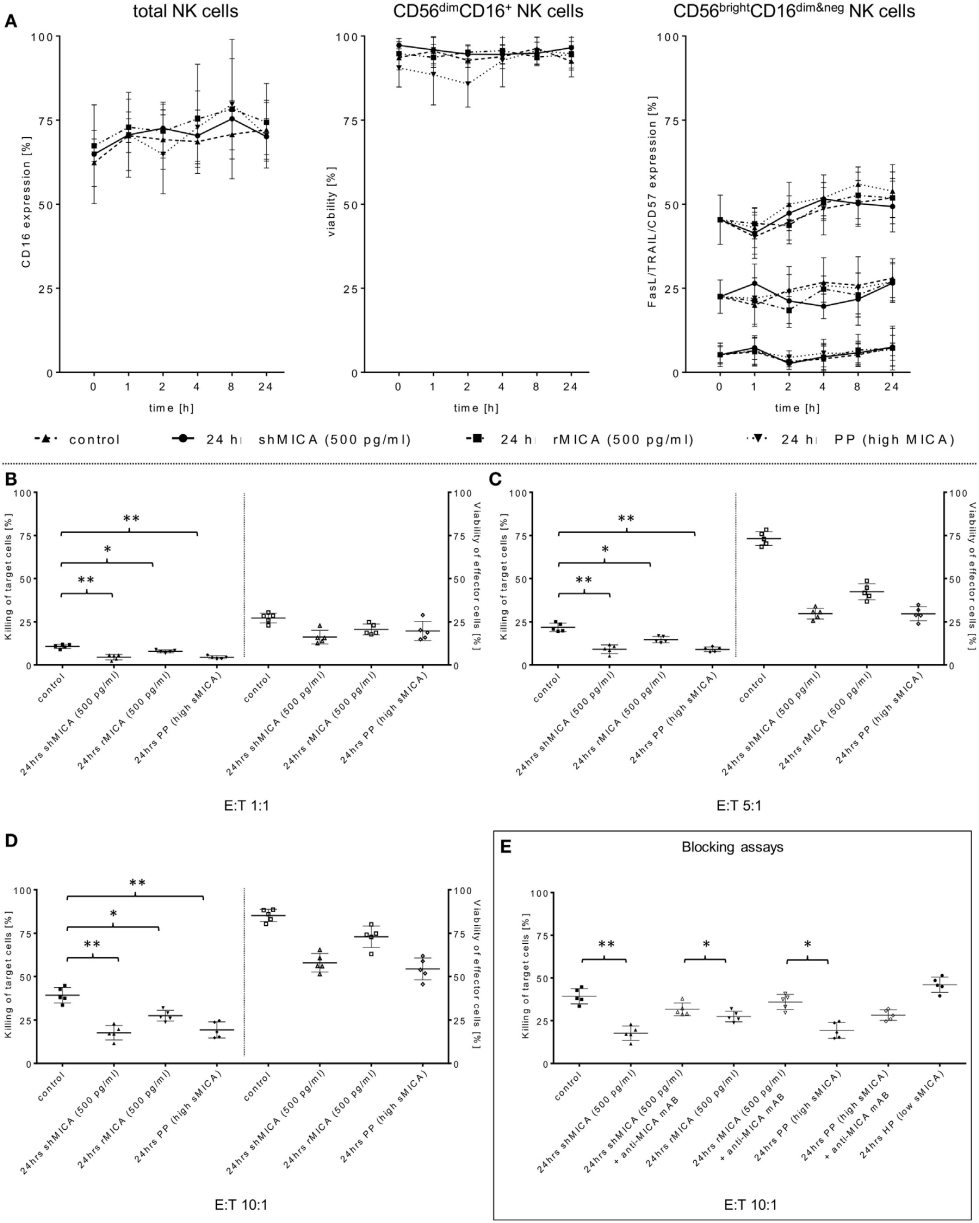
**Determination of the sMICA impact on ADCC-stimulating and pro-apoptotic NK cell receptors**. **(A)** Surface expression levels of CD16, TRAIL, FasL, and CD57 on the same effector cell samples were measured at the indicated time points and compared to non-treated control cells. As an additional control, effector cell viability was monitored over a 24-h period **(B–D)**. The impact of sMICA on the NKG2D-mediated NK cell cytotoxicity and NK cell stability during effector-target interactions against primary HNSCC cells (*n* = 5) was analyzed by FCM. Therefore, the same overnight-treated NK cell samples (see above, section Figure 2A) were subsequently co-incubated for 4 h (37°C, 5% CO_2_) with corresponding patient HNSCC target cells at the indicated E:T ratios. **(E)** Inhibition of sMICA effects on NKG2D-mediated NK cell cytotoxicity. SMICA molecules in all treatment samples (shMICA, rMICA, and PP) were blocked by pre-incubation (20 min) with MICA-specific mABs (20 μg/ml, MAB13001). NKG2D-dependent killing rates of those incubated NK cells against primary HNSCC cells were measured after 4 h (“Blocking assays,” ratio: 10:1, 37°C, 5% CO_2_) as described above. Data are shown as mean ± SD from three to four experiments for each patient. Statistically significant difference: *p ≤ 0.01 and **p ≤ 0.001.

**Figure 4:**
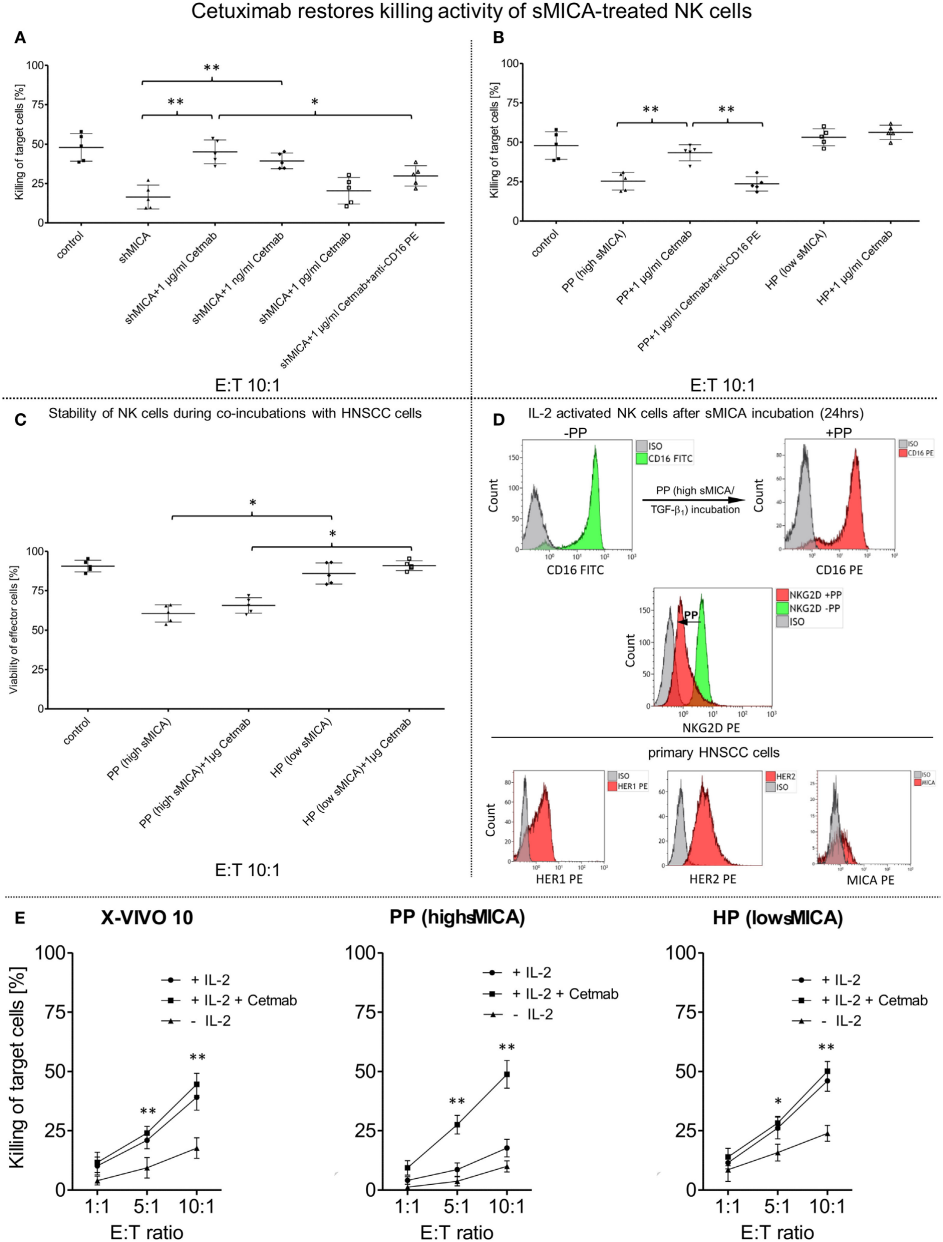
**Restored killing activity against primary HNSCC cells mediated by cetuximab**. IL-2 activated patient NK cells (*n* = 5) were incubated for 24 h (37°C, 5% CO_2_) with **(A)** shed MICA (shMICA, 500 pg/ml), **(B)** with patient plasma containing high MICA levels (PP, *n* = 5, range: 220.9–870.7 pg/ml) and healthy plasma (HP, *n* = 5, range: 1.9–28.5 pg/ml) served as a control. **(A)** CD16-mediated ADCC of those treated patient NK cells was assessed by FCM-based cytotoxicity assay using cetuximab (Cetmab, range of dose titration: 1 μg/ml–1 pg/ml)-coated primary HNSCC cells and compared to killing activity of shMICA-treated NK cells against non-coated HNSCC cells. **(B)** To overcome sMICA effects against NK cell cytotoxicity, 1 μg/ml cetuximab was chosen based on the earlier titration experiments (see above). Inhibition of cetuximab-dependent ADCC was achieved by blocking CD16 epitopes on treated patient NK cells with anti-CD16 mAb [20 μg/ml, 20 min pre-incubation, graphs **(A,B)**]. **(C)** The effector cell stability of PP- and HP-treated viable NK cells during tumor cell lysis was analyzed in presence and absence of 1 μg/ml cetuximab and compared to non-treated effector cell controls quantified by single platform functionality assays. **(D)** FCM-based characterization was utilized to monitor CD16 and NKG2D surface expression patterns on treated NK cells (CD16/NKG2D, see overlay histograms) before (−PP) and after (+PP) overnight incubation with patient plasma (PP) containing high sMICA and TGF-β_1_ levels. Analogously, HNSCC cell clusters were singularized for FCM-based characterization of target cell antigens (HER1/HER2/MICA) to assess alterations in the killing activity from different treated patient NK cells. **(E)** Effect of IL-2 on the killing activity of patient NK cells (*n* = 5) in presence (+IL-2/+Cetmab) and absence (+IL-2) of 1 μg/ml cetuximab (Cetmab). IL-2 expanded NK cells were incubated with patient plasma containing high MICA levels (PP, *n* = 5, range: 220.9–870.7 pg/ml), healthy plasma (HP, *n* = 5, range: 1.9–28.5 pg/ml) and control medium. Cytotoxicity of treated NK cells was analyzed at the indicated ratios with and without Cetmab (1 μg/ml)-coated primary patient HNSCC cells and compared to killing activity of unstimulated NK cells against non-coated HNSCC cells (−IL-2). **(A–D)** Data represent the mean ± SD of three experiments for each patient. Statistically significant difference: *p ≤ 0.01 and **p ≤ 0.001.

To analyze the NK cell-mediated killing activity in presence and absence of cetuximab, we developed a matched effector-target cell system based on a FCM-based cytotoxicity assay. Therefore, we utilized only concordant patient NK cells, patient plasma (PP, high sMICA) and primary patient HNSCC tumor cells (*n* = 5). To demonstrate the sMICA-mediated inhibition, IL-2 expanded (9–12 days) primary patient NK cells were co-incubated overnight (24 h, 37°C, 5% CO_2_, 250 rpm) with 500 pg/ml shMICA, PP containing high MICA levels (PP, range: 220.9–870.7 pg/ml) and, as a comparative control, with HC plasma (HP, range: 2.8–22.0 pg/ml) diluted 1:2 in X-VIVO™10 medium (Biowhittacker™Cambex Bioscience, Belgium). Phenotypical cell characterizations were accomplished to detect altered expression patterns of CD16 and NKG2D on treated NK cells (Figures 2 and 3). Singularized HNSCC cells were also tested for MICA, HER2, and HER1 surface expression, the latter as a target for restored ADCC before initiation of described cytotoxicity assays. To determine the effect of cetuximab on NK cell-dependent killing activity, various cetuximab doses (Cetmab: 1 pg/ml–1 μg/ml) were used to coat the corresponding HNSCC target cells (E:T ratio: 10:1). To inhibit putative effects of cetuximab toward NK ­cell-mediated cytotoxicity, the NK cells were pre-incubated for 20 min with anti-CD16 mAb (20 μg/ml). The effector-based ­cytotoxicity of these treated NK cell samples were analyzed against corresponding patient HNSCC cells in the indicated effector-to-target ratios (Figures 3 and 4). To avoid effector and target cell sedimentations or insufficient stirring of our co-incubated approaches during the cytotoxic reactions, the co-cultured cell suspensions were shaken in an CO_2_-incubator (CO2cell, 170-400 Plus, RS Biotech, Scotland) for 4 h (37°C, 5% CO_2_, 250 rpm). An optimized gating panel (Figure S1 in Supplementary Material) based on a no-wash single platform FCM procedure (FC500, Beckman Coulter, Germany) was applied. Treated NK cells were stained with several monoclonal antibodies (mAbs): CD45 FITC (fluorescein isothiocyanate), CD56 PE (­phycoerythrin) or NKG2D PE, CD16 PC-7 (phycoerythrin-cyanin-7) in order to exclude the effector cells from primary HNSCC target cells stained with CD9 FITC, CD9 PE, HER1 PE, MICA PE, or CD81 PE. Effector and target cells were stained with mAbs as described previously (47, 48). Target cell elimination by effector cells was calculated as the total loss of viable HNSCC target cells as follows (48, 49):

$$\text{Killing activity}=\left(\text{1}-{\text{ concentration}}_{\text{co-cultures HNSCC cells}/\text{μL}}/{\text{concentration}}_{\text{HNSCC control cells}/\text{μL}}\right)\times 100\%$$

### Fluorescence Microscopy

**Figure 5:**
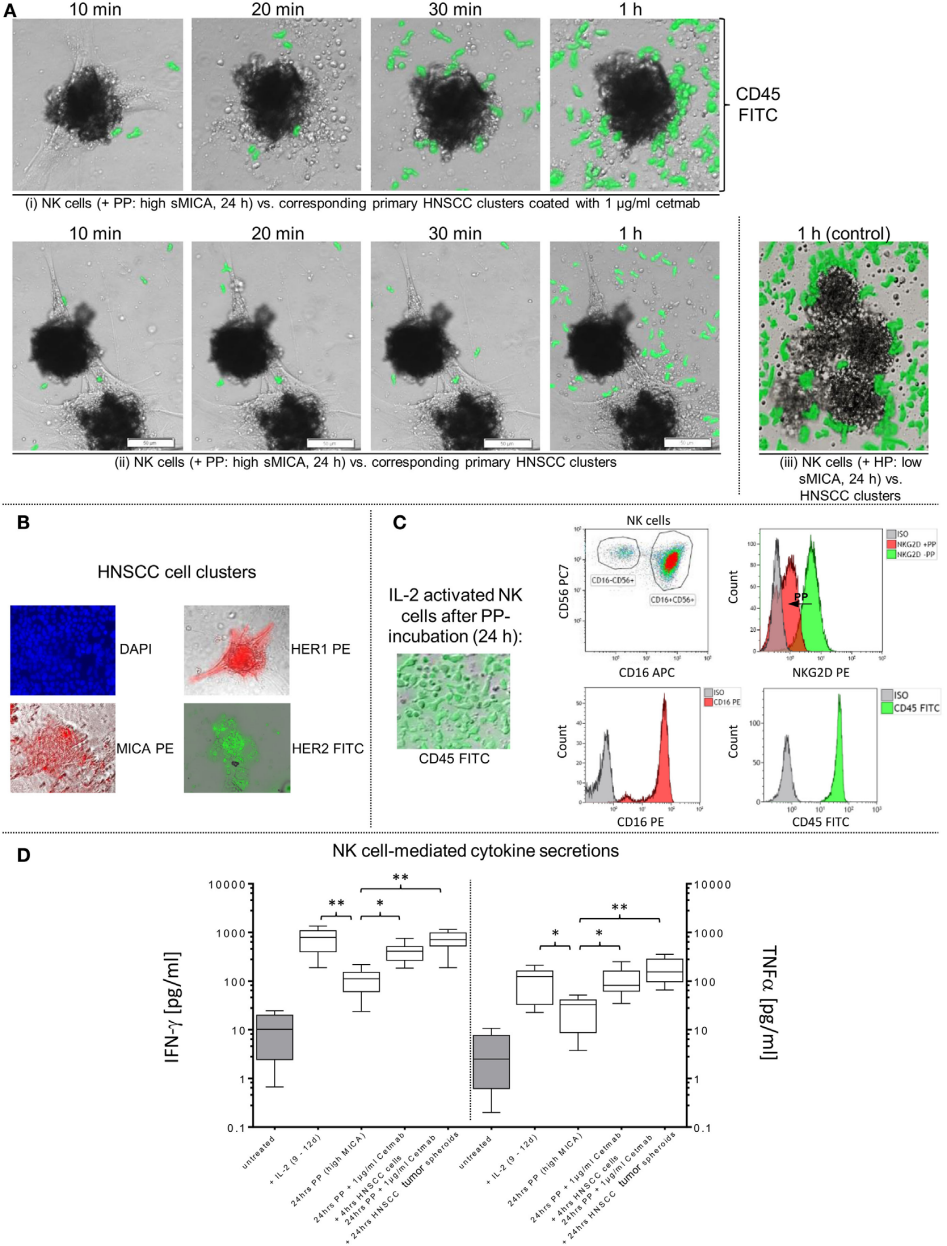
**Restored effector-target cell interactions via cetuximab-dependent ADCC of sMICA-inhibited NK cells**. **(A)** Activated patient NK cells were cultured (24 h) with patient plasma (PP; 1:2 diluted with X-VIVO™10) containing high sMICA levels and co-incubated (1 h, approximate E:T ratios of 5:1) with corresponding HNSCC cell clusters (time of cultivation: 3–12 days) in (i) presence and (ii) absence of 1 μg/ml cetuximab. (iii) Activated patient NK cells treated for 24 h with healthy plasma (HP [low sMICA]; 1:2 diluted with X-VIVO™10) were used to monitor early effector-target cell interactions, served as a positive control. CD45 (green) for PP- and HP-treated patient NK cells were analyzed by staining with FITC-conjugated mAb, whereas co-incubated adherent HNSCC clusters were unlabeled. Before initiating these functional assays, effector and target cells were tested flow cytometrically for relevant surface markers, especially MICA, HER2 and HER1 on HNSCC cells **(B)**, and CD16 and NKG2D levels on PP (high sMICA/TGF-β_1_)-treated NK cells **(C)**. Variable IFN-γ and TNFα secretions levels during co-incubations of treated NK cells with HNSCC cell clusters or tumor spheroids in presence or absence of cetuximab were quantified and compared with untreated controls **(D)**. Statistically significant difference: *p ≤ 0.01 and **p ≤ 0.001.

**Figure 6:**
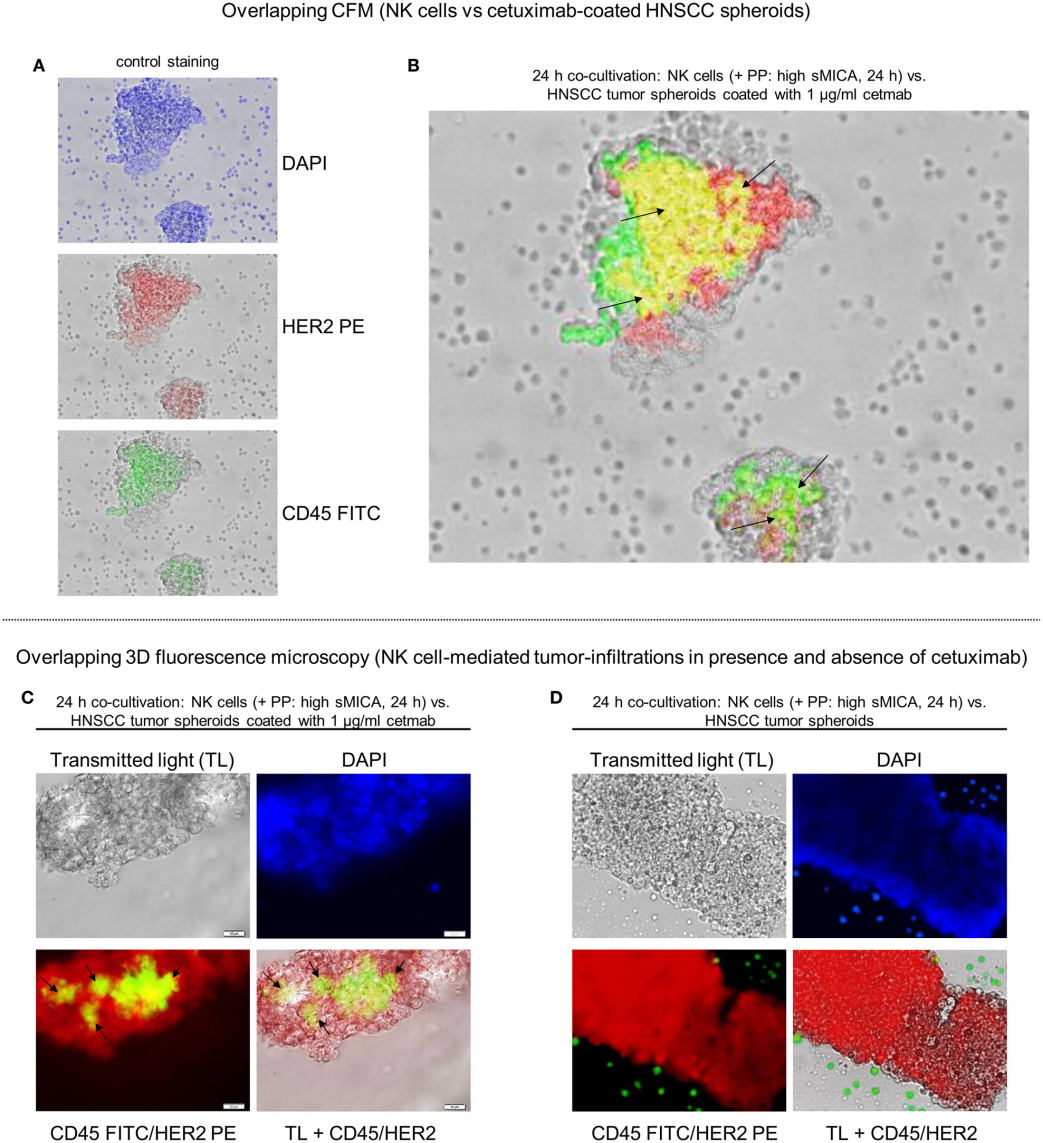
**Reconstitution of NK cell-dependent tumor infiltration by cetuximab**. Tumor recognition and infiltration of sMICA-affected NK cells in absence or presence of cetuximab (1 μg/ml) were analyzed by co-culturing (24 h, 37°C, 5% CO_2_) of corresponding patient NK cells, plasma (high sMICA) and HNSCC tumor spheroids derived from HNSCC patient tumors (*n* = 5). Activated NK cells were pre-incubated (24 h) with corresponding patient plasma containing high sMICA (PP: > 500 pg/ml; diluted 1:2 with X-VIVO™10) and co-incubated (24 h) with primary HNSCC spheroids (time of cultivation: 11–14 days) in the presence of 1 μg/ml cetuximab. CD45 (green) for PP-treated patient NK cells were identified by staining with FITC-conjugated mAb, whereas tumor spheroids were labeled with anti-HER2 PE-conjugated mABs **(A)**. Tumor-infiltrating NK cells were illustrated by overlay plots (CFM). Exactly the same orientation of CD45^+^ NK cells (green) and HER2^+^ tumor spheroids (red) showed a positive, overlapping signal in yellow [see black arrows **(B)**] for ADCC-related effector-target cell interactions, whereas single positive, HNSCC cells (CD45^−^/HER2^+^) are shown as red signals and single positive NK cells are depicted by green signals in the corresponding photographs **(A)**. 3D fluorescence microscopy visualized tumor-infiltrating patient NK cells (green-yellow areas) in corresponding HNSCC tumor spheroids in the presence **(C)** and absence **(D)** of 1 μg/ml cetuximab. DAPI [blue signals **(A,C,D)**] was used to stain DNA from methanol-fixed effector and target cells for analyses of the nuclear morphology.

IL-2 activated patient NK cells were cultured (24 h) with PP [(high sMICA); 1:2 diluted with X-VIVO™10] or healthy plasma [HP (low sMICA); 1:2 diluted with X-VIVO™10]. Afterward, these treated NK cells were co-incubated (1 h, 37°C, 5% CO_2_, approximate E:T ratios of 5:1) on 4-well chamber slides (1.7 cm^2^ growth area/well, 0.5–1.0 ml working volume, Nunc™, USA) with corresponding adherent HNSCC cell clusters derived from HNSCC patients (*n* = 5, time of cultivation: 3–12 days) in presence or absence of 1 μg/ml cetuximab for indicated periods of time (Figure 5A). The capability of tumor infiltration from those treated patients NK cells (see above) in corresponding patient HNSCC tumor spheroids (*n* = 5) were assessed with and without 1 μg/ml cetuximab and analyzed after 24 h co-cultivation (37°C, 5% CO_2_) (Figure 6). 2D/3D confocal fluorescence microscopy (CFM) using FITC- and PE-conjugated mAbs was used to separate stained patient NK cells (CD45 FITC or NKG2D PE) and corresponding HNSCC cell clusters or tumor spheroids (CD9 FITC, HER1 PE, HER2 PE, or MICA PE), as described before (47).

### Time-Lapse Microscopy

Transmission microscopy was used to monitor infiltration of sMICA-inhibited NK cells into corresponding HNSCC tumor spheroids. Low numbers of tumor spheroids were grown scattered on chamber slides for 16 h (37°C, 5% CO_2_). Subsequently, HNSCC spheroids were co-incubated with freshly isolated, non-stimulated patient NK cells (1 × 10^6^ effector cells/ml). Putative effector cell migration and tumor chemoattraction were monitored by time-lapse microscopy and imaging (Video S1 in Supplementary Material) as described previously (47).

### Blocking Assays

To inhibit the restored NK cell-based cytotoxicity by cetuximab, patient NK cells were pre-incubated for 20 min with anti-CD16 mAb (clone 3G8, 20 μg/ml, A07766, Beckman Coulter, Germany). Afterward, killing activity of treated NK cells was analyzed with cetuximab-coated primary patient HNSCC cells over a time period of 4 h (37°C, 5% CO_2_, 250 rpm) at an E:T ratio of 10:1 and compared to corresponding controls (Figure 4) as described previously (47). To analyze the direct role of sMICA on NKG2D-mediated cytotoxicity, we incubated IL-2-activated patient NK cells with shMICA, rMICA, and PP containing high sMICA levels overnight in the presence or absence of specific MICA antibodies, respectively (20 μg/ml, MAB13001, R&D systems, Germany). Additionally, plasma from five healthy donors (low sMICA, see above) was utilized to incubate patient NK cells as a control.

### Statistical Analyses

The Mann–Whitney non-parametric *U*-test was utilized to compare clinical and pathological parameters of plasma sMICA and TGF-β_1_ levels from HNSCC patients (*n* = 5) with healthy individuals (*n* = 5). The Student’s *t* test was used to assess the significance of the killing activity of patient NK cells incubated under various conditions. A *p* level ≥0.01 was considered statistically as non-significant. Unless otherwise declared, results of statistical evaluations from functional assays are indicated as mean ± SD and represent three to four experiments for each patient.

## Results

### Characterization of Altered NK Cell Subsets and Expression of NCRs in HNSCC Patients

Compared to age-matched healthy individuals (50), HNSCC patients showed a broad range of leukocyte subpopulations and absolute numbers of lymphocytes and leukocytes (Table 1). Although median NK cell amounts (12.8%; range: 2.7–33.2%) did not differ from HCs (Table 1), the absolute NK cell numbers (cells/μl) differed widely in the peripheral blood (PB) of patients and healthy donors (left graph sector, Figure 1A). Moreover, the proportion of immunoregulatory NK cells (CD56^bright^/CD16^dim&neg^) was markedly reduced in all patients [median: 2.4% (HNSCC_NK cells_) versus 11.8% in healthy donors (HD_NK cells_), middle graph sectors, Figure 1A]. In contrast, the cytotoxic NK cell subpopulation (CD56^dim^/CD16^+^) was strongly increased for all investigated HNSCC patients [median (HNSCC_NK cells_): 96.2% versus 86.8% (HD_NK cells_), middle graph sector, Figure 1A]. Moreover, freshly isolated patient NK cells revealed low to moderate expression levels of the NCRs, NKp30, NKp44, NKp46, and NKG2D compared to higher frequencies of IL-2 stimulated NK cells from HCs (right graph sector, Figure 1A). Nevertheless, the percentage of NK cells expressing NCRs increased (~4.7-fold, 3.8-fold, and 2-fold for NKp30, NKp44, and NKp46, respectively) during IL-2 activation over 9–12 days and was accompanied by ~60.7-fold higher expression levels of NKG2D (Figure 1A, right graphs) for all stimulated patient NK cells. The distribution of NK cell subpopulations shifted to higher CD56^bright^/CD16^dim&neg^ NK cell subsets (median before IL-2: 2.4% versus median after IL-2: 12.5%) and consequently lower percentages of CD56^dim^/CD16^+^ NK cells (median before IL-2: 96.2% versus median after IL-2: 88.9%) (Figure 1A).

### Reduced NK Cell-Dependent Cytotoxicity and Increased Immunosuppressive Factors in HNSCC Patients

The well-defined immunosuppressive factors sMICA and TGF-β_1_, which are responsible for impaired immunosurveillance, were quantified in PB from our HNSCC patients. Higher levels of both soluble factors were detected in HNSCC patients compared to HCs (sMICA, median: 532.8 versus 5.9 pg/ml; TGF-β_1_, median: 48.9 × 10^4^ versus 10.9 × 10^4^ pg/ml, respectively) (Figure 1B). Healthy plasma samples showed sMICA and TGF-β_1_ levels close to the detection limits of this assay [sMICA (mean ± SD): 10.8 ± 11.2 pg/ml; TGF-β_1_: 9.5 ± 5.2 × 10^4^ pg/ml; Figure 1B, HP], whereas sMICA (TGF-β_1_) in PP ranged between 220.9 and 870.7 pg/ml (25–64.8 × 10^4^ pg/ml) (Figure 1B, PP). To compare the basic killing activity between patient NK cells and NK cells from healthy individuals, freshly isolated, non-stimulated NK cells from both, patients and HCs were co-incubated overnight (37°C, 5% CO_2_) with corresponding HNSCC PP (high sMICA/TGF-β_1_) or associated HC plasma (low sMICA/TGF-β_1_). In both cases, the NK cell-mediated cytotoxicity was analyzed against the target cell line SCC-4 at the indicated ratios (4 h, 37°C, 5% CO_2_, 250 rpm) by FCM (Figure 1C). HNSCC plasma-treated patient NK cells (HNSCC, Figure 1C) exhibited significantly reduced cytotoxicity in all prepared E:T ratios when compared to NK cells from healthy donors pre-incubated with the corresponding HC plasma (Figure 1C).

### Phenotypical Analysis of Tumor-Relevant Expression Markers on Primary Patient HNSCC Cells

Expression levels of relevant surface antigens, especially HER1 and HER2, were examined on single tumor cell suspensions prepared from primary tumors surgically removed from HNSCC patients (*n* = 5, see above). Pre-characterized SCC-4 cells were utilized as a positive control for HER1 and HER2 expression because it was previously shown that these HNSCC cells displayed high amplification rates of HER1 genes in combination with enhanced HER2 and MICA expression levels (51). In addition to the HER1/2 levels, we analyzed expression levels of surface markers CD9 and MICA on the mono-dispersed patient tumor cells (Figure 1E). Representative overlay plots (FCM) were shown exemplarily in Figure 1E for prepared tumor cells derived from one HNSCC patient and were compared with qualitative evaluations determined by CFM. In Figure 1D, representative results of overlapping CFM exemplarily illustrated for cultured (1–2 days) adherent patient tumor cells after fluorescence labeling with similar stimulation energy and duration are shown. For counterstaining, we utilized DAPI as a fluorescent stain that binds strongly to A–T rich DNA regions of nuclei and chromosomes and emits blue fluorescence (Figure 1D). All tumor samples showed higher HER1 and CD9 levels in contrast to low-to-moderate expression of membrane-associated MICA and HER2 molecules. In summary, the antigens exhibited variable expression levels on all tumor samples of HNSCC patients and were slightly lower than on the SCC-4 control line as presented in Table 2.

**Table 2:** **Antigen expression levels measured on singularized patient HNSCC cells (*n* = 5) after preparation from tumors**.

	SCC-4	Patient 1	Patient 2	Patient 3	Patient 4	Patient 5
HER1	+++	++	+++	++	+++	++
HER2	+++	+/−	+/−	+	+	+
MICA	++	+/−	+	+/−	+	+
CD9	+++	++	++	++	+	++

*Fluorescence labeling of these tumor cells were compared to the SCC-4 cell line and intensities were scored as follows: weak (+/−), moderate (+), good (++), and strong (+++)*.

Primary patient HNSCC cells (*n* = 5) were cultured over 2 weeks (37°C, 5% CO_2_) to investigate time-dependent accumulation of immunosuppressive factors (sMICA and TGF-β_1_), and cell culture supernatants were collected at the indicated time periods (Figure 1F). A time-dependent increase of both immunosuppressive molecules was identified in cell culture supernatants in which the mean of sMICA levels increased from 18.7 ± 9.5 to 218.7 ± 131.9 pg/ml, and the mean of TGF-β_1_ levels increased from 6.8 ± 4.8 × 10^4^ to 40.9 ± 11.6 × 10^4^ pg/ml between the first and last time points monitored (Figure 1F).

### SMICA Affects NKG2D Expression and Cytotoxicity of IL-2-Activated Patient NK Cells

Tumor-derived TGF-β_1_ potentiated the sMICA-mediated down-regulation of NKG2D surface expression on various effector cells, especially NK cells, and resulted in decreased NKG2D-dependent immunity, thus reflecting the predominant role of the sMICA-NKG2D system (20, 52). Therefore, we co-cultured IL-2-activated patient NK cells overnight (24 h) with sMICA analogs (shMICA and rMICA, each in 500 pg/ml) and PP containing high sMICA levels (range: 220.9–870.7 pg/ml). All incubated NK cell samples from the five HNSCC patients exhibited a time-dependent down-regulation of NKG2D expression on total NK cells and both NK cell subsets (Figures 2A,B), whereas surface expression of CD16, pro-apoptotic FasL, and TRAIL receptors as well as activation marker CD57 were largely unaffected and stable over the indicated time period on all NK cell fractions by the described sMICA analogs (Figure 3A). We detected a distinct effect of shMICA on NKG2D expression on total NK cells and resultant NK subpopulations analyzed during the indicated time frame of 24 h (37°C, 5% CO_2_) by FCM. The time-dependent NKG2D down-regulation is displayed in plots exemplarily shown for one experiment (Figure 2B). Although surface phenotype, cell proportions, and viability of NK cells were not affected by co-incubation with PP (24 h, high MICA, Figure 3A), the observed decrease of NKG2D surface expression was accompanied by reduced IFN-γ and TNFα secretion compared to IL-2 activated patient NK cells [IL-2 (9–12 days), Figure 5D].

Additionally, the impact of sMICA on the NKG2D-mediated NK cell cytotoxicity of overnight co-incubated NK cells was analyzed against corresponding primary tumor cells derived from HNSCC patients (*n* = 5) to assess the degree of killing activity. Effector and target cells were co-incubated for 4 h (37°C, 5% CO_2_, 250 rpm) at indicated E:T ratios and killing activity was subsequently compared to X-VIVO™10-incubated controls without sMICA analogs. The cytotoxicity of shMICA- and PP-treated NK cells was strongly suppressed compared to low inhibition frequencies of rMICA-incubated effector cells at all E:T ratios (Figures 3B–E). During the cytotoxicity assays of co-cultured effector and target cells, the NK cell viability was decreased in shMICA-, rMICA-, and PP-treated NK cells as compared to more stable effector cells in X-VIVO™10-incubated controls (Figures 3B–E). To confirm the direct sMICA impact on the NKG2D-mediated NK cell cytotoxicity, MICA-specific mAbs (20 μg/ml, MAB13001) were used to deplete sMICA molecules in different treatment mixtures (shMICA, rMICA, and PP) (Figure 3E). NK cell cytotoxicity of these treated effector cells co-incubated with corresponding primary HNSCC cells were compared with sMICA analog-treated NK cells co-cultured without sMICA-specific mAbs as a negative control. The diminished killing activity of sMICA-affected NK cells was partially restored when compared to untreated (X-VIVO™10) control effector cells and to NK cells pre-treated with healthy plasma (HP, low sMICA) (Figure 3E).

### Cetuximab Restores NK Cell-Dependent Cytotoxicity Against Primary HNSCC Cells via ADCC

Cetuximab is a therapeutic mAb directed against the HER1 epitopes on several types of high-malignant tumors (53). Consequently, cetuximab is a powerful stimulus of NK cell-mediated ADCC via activation of FcγRIIIa against cetuximab-coated tumor cells and for induction of cytokine release, especially IFN-γ and TNFα secretion. Figure 4A demonstrates significantly restored NK cell-based cytotoxicity against cetuximab-coated HNSCC cells with mAb concentrations of 1 μg/ml (mean: 43.8 ± 10.8%) and 1 ng/ml (mean: 38.4 ± 6.8%) as compared to reduced killing activity of shMICA-incubated NK cells (mean: 13.4 ± 11.2%) against non-coated HNSCC cells and anti-CD16 mAb-blocked NK cells (mean: 28.8 ± 8.9%). Based on these titration experiments, only 1 μg/ml cetuximab were applied in subsequent experiments to restore the decreased NK cell cytotoxicity (mean: 41.5 ± 6.3%) after overnight incubation with PP (high sMICA, mean of cytotoxicity: 26.1 ± 6.3%) and blocking experiments with anti-CD16 mAb-treated NK cells (mean of cytotoxicity: 24.8 ± 5.7%) (Figure 4B). Untreated (X-VIVO™10) and healthy plasma (HP, low sMICA)-incubated patient NK cells were defined as unaffected control cells. NK cell viability was affected exclusively by PP-treatment and HP-incubated NK cells exhibited similar viability levels as untreated effector cells (Figure 4C). As internal effector cell controls, we assessed the activation marker NKG2D and IgG Fc receptor (CD16) of PP-treated (high sMICA) NK cells after 24 h overnight incubation by FCM. As shown in Figure 4D (upper row), the expression of NKG2D was significantly decreased but the CD16 expression levels were not changed on these effector cells. Otherwise, the phenotypical characterizations of target cell parameters showed higher levels of HER1 and HER2 antigens and only low-to-moderate MICA expressions on corresponding patient HNSCC cells (Figure 4D, lower row).

To determine whether IL-2 potentiates the cetuximab-mediated outcome on restored effector cell cytotoxicity against HNSCC cells, we analyzed the degree of ADCC from X-VIVO™10-, PP-, and HP-treated patient NK cells against cetuximab-coated and non-labeled HNSCC cells in the presence or absence of IL-2 (Figure 4E). Reconstituted NK cell cytotoxicity via ADCC was detected against cetuximab-coated HNSCC cells (IL-2 and Cetmab) compared to decreased cytotoxicity of PP-incubated NK cells against non-labeled HNSCC cells independently from the presence (+IL-2) or absence of IL-2 (Figure 4E, middle graph). Combination of IL-2 with cetuximab-coated or non-coated HNSCC cells (IL-2 with Cetmab or IL-2 alone) revealed no improvement of ADCC from X-VIVO™10- and HP-treated NK cells, but inclusion of IL-2 significantly enhanced NK cell cytotoxicity compared to untreated NK cells (**-**IL-2) (Figure 4E, left/right graphs). The cetuximab-mediated reconstitution of NK cell cytotoxicity correlated with higher IFN-γ and TNFα secretion levels (24 h PP + 1 μg/ml Cetmab + 4 h HNSCC cells) compared to PP-treated NK cells [24 h PP (high MICA) Figure 5D].

### Cetuximab Reconstitutes NK Cell Infiltration into HNSCC Clusters and Tumor Spheroids

To assess the capability of tumor infiltrations from sMICA-affected patient NK cells in absence or presence of 1 μg/ml cetuximab, we developed different *in vitro* models by establishing co-cultures (37°C, 5% CO_2_). Therefore, activated patient NK cells were pre-incubated overnight with corresponding PP (high sMICA) or healthy plasma (HP, low sMICA) served as a positive control. Afterward, the early recognition and tumor-infiltration capabilities of these treated effector cells were monitored after 1 h co-incubation with primary HNSCC cell clusters (Figure 5) or after 24 h in HNSCC tumor spheroids derived from five HNSCC patients to identify specific tumor-infiltrated NK cells (Figure 6).

Early “effector-to-target” affinities of treated NK cells against adherent HNSCC cell clusters coated or not coated with 1 μg/ml cetuximab were monitored (10 min–1 h) by CFM. The experiments showed clearly impaired and disordered “effector-to-target” interactions and decreased HNSCC cell cluster infiltrations from PP-treated NK cells in absence of cetuximab (Figure 5A, one row) compared to normal tumor infiltration capabilities of HP-treated (low sMICA) NK cells, served as a positive control [Figure 5A, lower row, right picture (control)]. In contrast, early effector cell-dependent infiltration within 20–30 min and restored anti-tumor reaction was observed by PP-treated NK cells against cetuximab-coated HNSCC clusters after 1 h (Figure 5A, upper row) accompanied by raised IFN-γ and TNFα medium levels (24 h PP + 1 μg/ml Cetmab + 4 h HNSCC cells, Figure 5D). Otherwise, the cytokine secretions of PP-inhibited NK cells against non-coated HNSCC cell clusters (without cetuximab) revealed significantly degraded IFN-γ and TNFα concentrations analyzed in medium supernatants [24 h PP (high sMICA), Figure 5D].

In addition, phenotypical analysis of relevant effector and target cell parameters were analyzed before co-cultivations. HNSCC cells revealed high HER1 and CD9 expression levels (not shown) and low-to-moderate MICA levels on the target cell clusters (Figure 5B). Otherwise, treated NK cells with corresponding PP (high sMICA, 24 h) showed a decreased NKG2D surface expression as detected by FCM analysis, while CD16 surface expression was unaffected (Figure 5C). In this context, sMICA and TGF-β_1_ accumulated time-dependently in the cell culture medium as summarized in Figure 1F analyzed for different time periods (0–2 up to 11–14 days).

Analysis of tumor-infiltration assays were also performed with PP-treated (high sMICA) NK cells co-cultured (24 h, 37°C, 5% CO_2_) against non-coated HNSCC tumor spheroids. As monitored for one spheroid by time-lapse imaging, effector-to-target cell interactions and tumor infiltrations of NK cells were strongly abolished and resulted in a lack of recognition of the HNSCC spheroid by non-functional NK cells, even though they were in close proximity to the spheroids (24 h, Video S1 in Supplementary Material). However, the PP-mediated inhibition of NK cell functions was also clearly restored by cetuximab-coated HNSCC spheroids (1 μg/ml cetuximab) via multiple effector-target interactions exemplarily shown for one representative overlay photograph (Figure 6B, right overlay plot containing yellow regions) generated by 2D CFM. Similarly, 3D fluorescence microscopy allowed visualization of the reconstituted specific tumor infiltrations by PP-treated (high sMICA) NK cells via putative ADCC in HNSCC tumor spheroids over 24 h in the presence of 1 μg/ml cetuximab (green-yellow areas, Figure 6C). Moreover, the medium supernatants of those incubated samples (PP-cultured NK cells and cetuximab-coated HNSCC tumor spheroids) revealed significantly increased IFN-γ and TNFα secretion levels (24 h PP + 1 μg/ml Cetmab + 24 h HNSCC tumor spheroids, Figure 5D). However, the tumor infiltration [CD45^+^ NK cells (green signals), Figure 6D] and cytokine release [24 h PP (high sMICA), Figure 5D] of same PP-inhibited NK cells were strongly reduced in 24 h-incubated samples in absence of cetuximab and consequently non-coated HNSCC tumor spheroids.

## Discussion

Dysfunctional tumor surveillance in HNSCC patients is further hampered by tumor immune escape mechanisms, which may induce dysregulation of immunocompetent cell profiles. In contrast to literature reports describing decreased NK cell numbers in HNSCC MNCs (54), we did not observe marked alterations in total NK cells among the total lymphocytes in our cohort of five relapsed HNSCC patients not currently in treatment. Previously, we demonstrated disbalances in NK cell subpopulations in 55 patients with initial and relapsed HNSCC (22). However, the immunoregulatory NK cell population responsible for stimulation of immature DCs by TNFα and IFN-γ secretion (5, 55) was significantly decreased in comparison with age-matched healthy individuals. Accordingly, Wulff et al. reported reduced regulatory NK cells in many HNSCC patients at different tumor stages (56). We detected higher levels of cytotoxic NK cell subpopulations compared to HCs, which may reflect suppressed cytotoxic interactions against HNSCC cells and limited tumor-infiltration capacities of these inhibited NK cell subsets. Additionally, we demonstrated that alterations in NK cell subtypes in our HNSCC patients were accompanied by disrupted TNFα and IFN-γ secretion. Enhanced sMICA and TGF-β_1_ plasma levels in HNSCC patients also correlated strongly with NKG2D-dependent dysfunction of patient NK cells, resulting in suppressed killing activity against HNSCC cells and decreased NK cell viability during cytotoxic effector-target interactions. In accordance, Bose et al. described marked alterations in the Th1/Th2 cytokine ratios and significantly increased suppressor regulatory T cells in cultured MNCs from HNSCC patients resulted in decreased cytotoxicity of HNSCC effector cells (54). According to our cytotoxicity experiments, high sMICA/TGF-β_1_ levels derived from HNSCC PP also contribute to diminished effector cell stability. This was also confirmed by Rossi et al. via correlation between impaired NK cell viability, effector cell cytotoxicity and decreased NKG2D and NKp46 surface expression (57). Increased NK cell susceptibility also reflects the ability of the tumor to release apoptosis-promoting factors (programmed death receptor ligand, PDL-1), which can abolish several effector cell functions within the tumor microenvironment as detected on PD-1^+^ NK cells in cancer patients (58, 59).

We translated an activation protocol from a previous phase I/II trial (Clin-Gov-No-NCT01386619), which described stimulation of allogeneic NK cells (1000 IU/ml IL-2) with resultant increased distribution of NK cell subsets and high NCR expression levels (11), to NK cells isolated from our HNSCC patients. After IL-2 stimulation, the activated patient NK cells revealed improved distribution of increased immunoregulatory NK cells (CD56^bright^/CD16^dim&neg^), enhanced TNFα and IFN-γ secretion and up-regulated NKG2D expression levels that resulted in enhanced cytotoxicity against associated HNSCC cells. Increased expression of CD56 was coincident with higher levels of NCRs as was also detected in other studies, suggesting that tumor-infiltrating NK cells were activated effector cells, but characterized by poor functionality (39, 60, 61). Indeed, our incubations (24 h) of activated NK cells with sMICA analogs or PP containing increased sMICA resulted in decreased NKG2D expression followed by impaired NKG2D-dependent killing activity against associated HNSCC cells. However, NK cell viability and expression levels of CD16, pro-apoptotic FasL and TRAIL receptors as well as for the activation marker CD57 were not affected by sMICA. In contrast, the significantly decreased levels of FasL on cultured HNSCC MNC NK cells shown by Bose et al. might be due to marked up-regulation of suppressor regulatory T cells followed by enhanced TGF-β_1_ secretion levels (54).

Our observations regarding inhibited cytotoxic functions of sMICA-treated NK cells due to suppressed HNSCC tumor infiltration indicate suppressed migratory capacity of NK cells toward HNSCC tumors as detected by transmitted time-lapse imaging (Video S1 in Supplementary Material). This is one of the numerous effector cell functionalities, impairment of which can result in TIEM allowing unhindered tumor growth. NKG2D is an activating receptor for NK, NKT, CD8^+^, and γδ^+^ T effector cells, and down-regulation of NKG2D in HNSCC patients seems to be a crucial mechanism of immune evasion. Soluble NKG2D ligands (NKG2DL) in association with growth factors, such as TGF-β_1_, released from mesothelioma cell-generated exosomes were described to potentiate the down-modulation of NKG2D surface expression on activated NK cells (62). Indeed, we observed significant correlations between time-dependent high sMICA and TGF-β_1_ secretion levels and increased HNSCC cell growth of our tumor clusters or spheroids, which also resulted in disrupted NKG2D-mediated immunosurveillance of blocked patient NK cells as shown previously in our HNSCC patient study (22). However, the negative impact of increased TGF-β_1_ levels detected in these HNSCC patients could be inhibited by neutralization antibodies against this immunosuppressive factor, indicating that TGF-β_1_ seemed to potentiate the sMICA-induced decrease of patient NK cell cytotoxicity and diminished NKG2D expression. According to our results, others have demonstrated restored NKG2D expression levels on NK and CD8^+^ T cells after tumor resection in glioma patients, which was accompanied by enhanced killing activities of those effector cells against NKG2DL-positive tumor targets (63). Interestingly, NKG2D was markedly down-regulated on activated CD8^+^ T cells but only if CD4^+^ T cells and NKG2DLs, such as soluble MICB, were present (64). This observation supports the hypothesis that soluble NKG2DLs played a secondary role and the down-regulation of NKG2D was primarily caused by tumor-derived TGF-β_1_ (63). The immunosuppressive tumor environment was responsible for the high diversity of antigen presentation patterns in stroma-infiltrating NK cells and tumor-infiltrating NK cells. The effector cells showed lower CD56 levels and higher CD16 expression during cytotoxic interactions with breast cancer cells and resulted in altered NK cell phenotypes with decreased functional capacities (65). In accordance with this report, we demonstrate stable CD16 expression levels, which were not affected by sMICA analogs or HNSCC plasma containing high amounts of sMICA and TGF-β_1_.

NK cell subsets have a stimulating Fc receptor for binding IgG (Fcγ RIIIa), which induces ADCC and may trigger TNFα and IFN-γ secretion to finally recognize and kill antibody-coated targets (66). Since several ErbB family members, including HER1 (EGFR), HER2 and HER3, seem to be strong predictors for the outcome of HNSCC (67), we stained different tumor samples post-surgery from corresponding relapsed patients for HER1/2 and collected PB for NK cell separation as well as blood plasma for quantification of sMICA and TGF-β_1_. The chimeric (human-murine) IgG_1_ cetuximab directly affects HER1-positive tumor cells by activation immunocompetent cells (66). Therefore, we assessed our sMICA-affected NK cells regarding to cytotoxicity and tumor infiltrations. We found improved killing activity via ADCC for sMICA-inhibited NK cells against cetuximab-coated HNSCC cells. Additionally, enhanced cytokine (TNFα and IFN-γ) release was observed compared to low cell lysis of the same effector cells co-cultured with untreated tumor cells. Negative effects of sMICA and TGF-β_1_ on NK cell cytotoxicity were overcome by cetuximab and correlated well with high IFN-γ and TNFα secretion levels described previously in HNSCC and other cancers (53, 68). In this context, it was reported that cetuximab-induced NK cells are able to activate DC maturation markers and antigen presentation machinery via IFN-γ secretion, thus allowing initiation of adaptive immune responses by NK cell stimulated DC maturation (69). We demonstrated that combining cetuximab with IL-2 (1000 IU/ml) re-established NK cell cytotoxicity and reconstituted TNFα and IFN-γ secretion. This is in agreement with other reports that described increased ADCC activity and cytokine release in the presence of cetuximab and several cytokines (IL-2, IL-12, IL-15, and IL-21) compared to cetuximab-mediated ADCC in absence of these cytokines (53, 65, 68). Prospective studies should clarify how to improve the cetuximab effect combined with pro-inflammatory cytokines, especially IL-2. Interestingly, it was demonstrated that ADCC- and IL-2-activated NK cells were less susceptible to immunosuppression by chemotherapy or other immunosuppressive drugs, especially mycophenolate mofetil (MMF), than non-stimulated NK cells in cancer patients (68, 70).

To investigate the potency of cetuximab on cytotoxic functionalities of sMICA-inhibited NK cells, we generated HER1-positive tumor spheroids to simulate the *in vivo* tumor microenvironment. This enabled us to analyze the restoration of tumor-infiltrating capability of NK cells with down-regulated NKG2D expression levels in a well-defined system. Tumor spheroids were utilized to monitor specific migratory capability and tumor chemoattraction of effector cells, especially NK cells, over well-defined time periods (71). However, only few reports describe the effector cell-mediated immunosurveillance toward tumor spheroids. Inactivation of NK cells by clustered Ewing’s sarcoma cells and cytotoxicity of γδ^+^ T cells against pediatric liver tumor spheroids was demonstrated (72, 73). In our tumor models, we were able to assess the cetuximab-mediated tumor infiltration from sMICA-inhibited NK cells toward corresponding primary HNSCC cell clusters and tumor spheroids that expressed high levels of HER1 and HER2. HNSCC spheroids expressed only low-to-moderate levels of membrane-bound MICA, but sMICA and TGF-β_1_ release steadily increased in supernatants of cultured HNSCC tumor spheroids and reached saturation levels after a few days of cultivation. The accumulation of immunosuppressive factors in HNSCC spheroid supernatants supported the assumption that NKG2D-mediated cytotoxicity of co-cultured patient NK cells was diminished, which corroborates the negative effect toward NK cell-mediated immunosurveillance by these tumor spheroids as described previously (71). Importantly, we detected early effector-to-target interactions displayed in small HNSCC tumor cell clusters during the first hours of co-cultivation in the presence of cetuximab. Furthermore, we successfully demonstrated cetuximab-mediated tumor infiltrations and increased TNFα and IFN-γ secretions of sMICA-inhibited NK cells in associated HNSCC spheroids using 2D- and 3D-microscopy techniques. This supported results from monolayer cultures of different effector-to-target cell ratios co-incubated for previous cytotoxicity assays. Correspondingly, others evaluated the direct localization of fluorescently stained therapeutic antibody, cetuximab-IRDye800CW, in histologic sections with tonsil, tongue, and cutaneous squamous cell carcinoma (SCC) by fluorescence immunohistochemistry (74). Interestingly, the heterogeneous composition and tumor architecture in short-term culture of HNSCC tumor slices demonstrated a high diversity of individual responses to cetuximab, but the absence of any effector cell subset (75). It was recently suggested that drug resistance to anti-EGFR therapies in HNSCC is not affected by the hypoxic tumor microenvironment within the investigated tumors (76). Indeed, the inhibition of EGFR via cetuximab reduces angiogenesis via hypoxia-inducible factor-1α and Notch1 in HNSCC (77).

## Conclusion

In conclusion, our current results emphasize that cetuximab is able to neutralize negative effects of TIEMs. This was shown in a corresponding effector-target system of patient NK cells, PP containing immunosuppressive factors (sMICA and TGF-β_1_), and associated HNSCC tumors/tumor cells as targets. This is of clinical relevance as the shedding of soluble NKG2DLs combined with secretion of immunosuppressive cytokines may promote tumor progression and is predictive for a negative prognosis in tumor patients (78).

In summary, observations of our clinical phase I/II haploidentical NK cell study for adaptive immunotherapy (Clin-Gov-No-NCT01386619) (47, 79, 80) and our current results indicate that allogeneic NK cells in combination with tumor antigen-specific mAbs, especially cetuximab, might be an innovative approach to circumvent TIEM-derived limitations against patient NK cells. Therefore, it is necessary to monitor overexpressed tumor antigens, such as HER1, and immunosuppressive ligands and the polymorphic Fcγ receptor IIIa to regenerate the tumoricidal properties of NK cells for improved therapeutic benefit.

## Conflict of Interest Statement

The authors declare that the research was conducted in the absence of any commercial or financial relationships that could be construed as a potential conflict of interest.

## Supplementary Material

Figure S1 **Gating strategy**. Flow cytometry-based cytotoxicity assay of NK cells against singularized HNSCC cells. NK and HNSCC cells were stained with CD9 FITC, CD56 PC7, and CD45 KO mAbs. 7-AAD (7-amino-actinomycin D) was used to discriminate non-viable cells in FCM. Blue: CD45+ NK cells; red: CD9+ HNSSC cells; and grey: 7-AAD+ effector and target cells. Dot plot areas were defined by the analysis of mono-cultured NK and HNSCC cells incubated for the identical time periods. Dot plot (WBC/MNC) displays an overview of all scatter events properties, and shows differentiations to non-specifically stained debris by low forward scatter signals. Plot (CAL) illustrates the events of the region “beads” along the time course to calibrate the events for “cells/μl” and detect even sample flow. Region (Target cells) is defined to include viable CD9+ HNSCC cells, and region (Effector cells) is defined to include all CD45+ NK cells. Plot (“Cell clusters”) represents the viable effector-target–cell interactions. Plot (“Viability”) is defined as a region to exclude the 7-AAD+ cells. These 7-AAD+ cells are not further presented in the following dot plots by using the characteristic signal when representing the 7-AAD fluorescence against the side scatter (SSC) properties.

Video S1 **Time-lapse imaging of PP (high sMICA and TGF-beta1 levels)-treated NK cells against corresponding HNSCC tumor spheroids over a time period of 24 h**. These NK cells (smaller rounded effector cells) isolated from the same HNSCC patient showed a decreased tumor recognition against associated tumor spheroids via reduced migratory capability and a decreased cytotoxicity.
