# Supplementary figures and images for: Cetuximab Reconstitutes Pro-Inflammatory Cytokine Secretions and Tumor-Infiltrating Capabilities of sMICA-Inhibited NK Cells in HNSCC Tumor Spheroids
Source: Front Immunol. 2015 Nov 2;6:543. doi: 10.3389/fimmu.2015.00543 (PMC4629470; doi:10.3389/fimmu.2015.00543)

## Cytotoxicity assay (gating strategy):

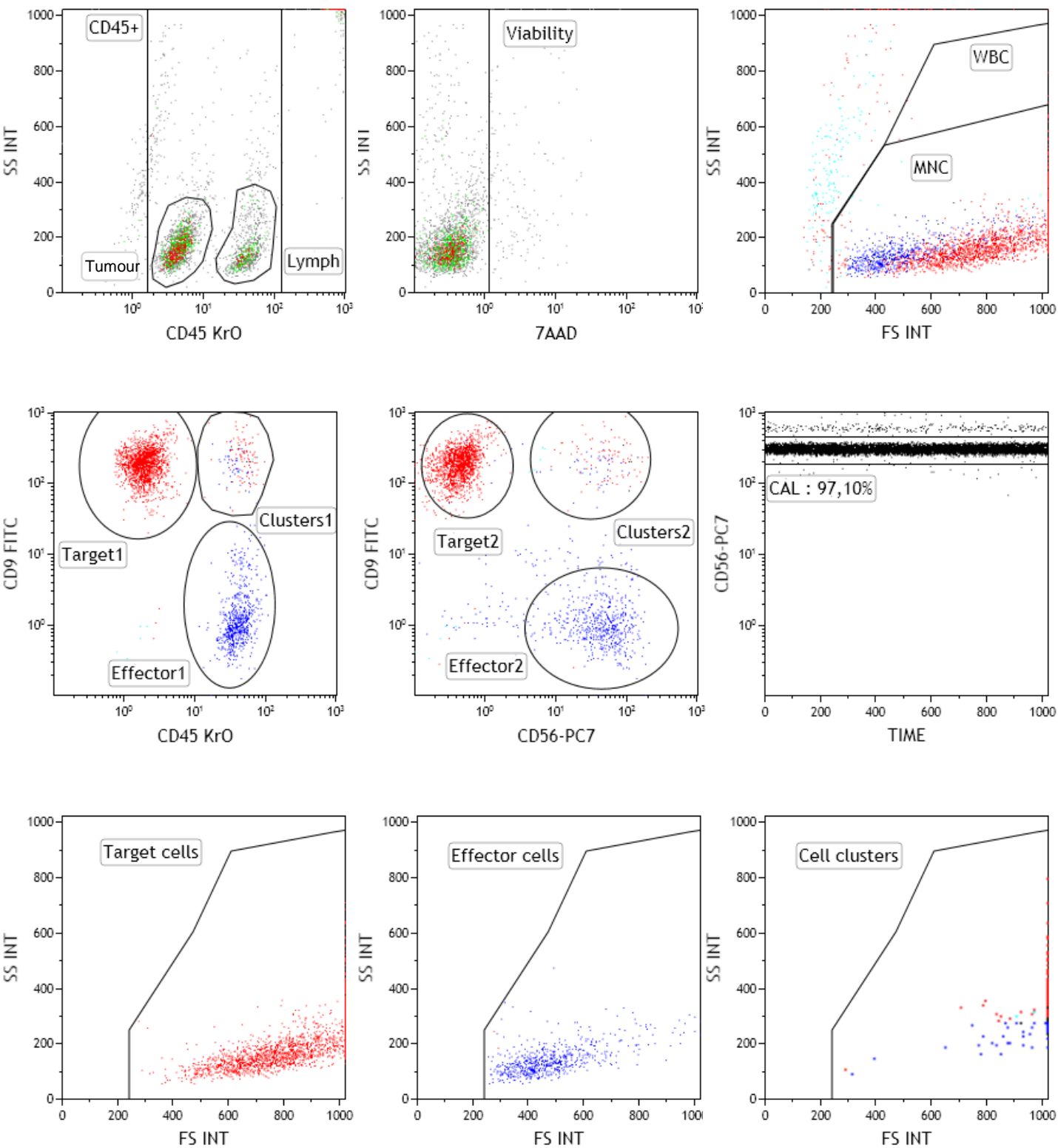

Supplement: Figure S1 — Gating strategy. Flow cytometry-based cytotoxicity assay of NK cells against singularized HNSCC cells. NK and HNSCC cells were stained with CD9 FITC, CD56 PC7, and CD45 KO mAbs. 7-AAD (7-amino-actinomycin D) was used to discriminate non-viable cells in FCM. Blue: CD45+ NK cells; red: CD9+ HNSSC cells; and grey: 7-AAD+ effector and target cells. Dot plot areas were defined by the analysis of mono-cultured NK and HNSCC cells incubated for the identical time periods. Dot plot (WBC/MNC) displays an overview of all scatter events properties, and shows differentiations to non-specifically stained debris by low forward scatter signals. Plot (CAL) illustrates the events of the region “beads” along the time course to calibrate the events for “cells/μl” and detect even sample flow. Region (Target cells) is defined to include viable CD9+ HNSCC cells, and region (Effector cells) is defined to include all CD45+ NK cells. Plot (“Cell clusters”) represents the viable effector-target–cell interactions. Plot (“Viability”) is defined as a region to exclude the 7-AAD+ cells. These 7-AAD+ cells are not further presented in the following dot plots by using the characteristic signal when representing the 7-AAD fluorescence against the side scatter (SSC) properties. [file image_1.pdf]
